# Food and mood: how clean eating content on social media influences affect and body satisfaction in women

**DOI:** 10.3389/fpsyg.2025.1531142

**Published:** 2025-09-22

**Authors:** Giulia Cossu, Alina Riefler, Andrea Sabrina Hartmann

**Affiliations:** Unit of Clinical Psychology and Psychotherapy of Childhood and Adolescence, Department of Psychology, University of Konstanz, Konstanz, Germany

**Keywords:** affect, body satisfaction, clean eating content, foodie content, Instagram

## Abstract

**Introduction:**

Body-related content is omnipresent in social media. Its consumption has shown to impact body image and affect. Clean eating content is often related to body-related content but its relation to the aforementioned constructs has not been examined.

**Methods:**

In an experimental study, 129 female individuals viewed clean eating or “foodie” content (control condition) on Instagram profiles for 5 min. Positive and negative affect, as well as body satisfaction were measured before and afterwards. We also assessed the behavioral choice of a food after profile viewing, and whether pre-existing vulnerabilities (body dissatisfaction, eating disorder or orthorexic symptoms) impact the change in affect and body satisfaction.

**Results:**

There was no significant profile × time interaction effect for positive affect, however it decreased over time (_p_𝜂^2^ = 0.21). Negative affect was reduced after foodie content consumption as opposed to the respective other profile (_p_𝜂^2^ > 0.03). An additional interaction effect in body satisfaction did not substantiate in simple main effects. Pre-existing vulnerabilities did not impact reactivity of individuals to CE content.

**Discussion:**

There was no impact of profile on choice of foods. The change in positive affect underlines previously shown potentially detrimental effects of Instagram consumption in general. The findings also give tentative hints toward the potential negative influence of clean eating content consumption on affect and body dissatisfaction. Thus, also a focus on clean eating might need to be incorporated when targeting media literacy in prevention programs.

## 1 Introduction

In recent years, (time and frequency spent on) social media has shown to be strongly associated with body satisfaction and disordered eating in its users (Rounsefell et al., 2020; Holland and Tiggemann, 2016; Mingoia et al., 2017; Rodgers and Melioli, 2016; Saiphoo and Vahedi, 2019). Particularly appearance-related social media use has been shown this association, more strongly so than general (Mingoia et al., 2017; Saiphoo and Vahedi, 2019) or communication-based social media use (Markey and Daniels, 2022). Given the prevalence of social media use, with 28% of the world’s internet users being registered on Instagram as one of the main picture-based social media outlets (Auxier and Anderson, 2021), it is essential to broaden the view on related imagery beyond body pictures.

Beyond, body-related imagery, a limited but emerging field of research has started to examine the impact of food- and eating-related social media posts on individuals’ perceptions of their bodies and eating behaviors. This line of inquiry is influenced by earlier studies on traditional media, which have indicated that certain media-related activities—such as television viewing—may contribute to higher energy consumption (e.g., Gore et al., 2003; Hetherington et al., 2006) and less healthy dietary patterns (e.g., Harris and Bargh, 2009). Social media might even amplify the spread of such content, especially when combined with the promotion of thinness and dieting ideals. It is worth noting that social media features a wide range of popular eating-related content, including food photography, nutritional tips, and eating videos. While there is considerably less empirical data in comparison to body-image related content, a systematic review brought together 38 studies investigating the relationship between eating-related content and factors such as body image, eating behavior, and viewer perceptions (Wu et al., 2024). The authors concluded that there seem to be links between consuming eating-related content and negative body image, eating disorder symptoms, and more inconsistent findings regarding the association between exposure and food choices or intentions to consume. The former conclusion is supported by another systematic review including 16 studies targeting the relationship of food-related social media content with body image and disordered eating, respectively (Roorda and Cassin, 2025).

One eating-related content trend is “clean eating” (CE), that has gained traction recently, with many thousand posts on Instagram containing the hashtags #healthylifestyle and #healthyfood (Pilař et al., 2021). While there is no universally agreed-upon definition of the phenomenon of CE, it can be described as an eating behavior characterized by the consumption of mainly non-processed foods (e.g., self-prepared and/or raw food, seeds) (Allen et al., 2018). These foods are compared to other ingredients (e.g., refined sugar, alcohol) which are deemed to be impure and should therefore be avoided, leading to a perceived categorization of food into “good” and “bad” foods, often associated with a strict diet (Walsh and Baker, 2020). While the concept of CE may be initially considered as beneficial to health, restrictions on both the quantity and/or variety of food can potentially develop into dietary restraint in 17–55 year-old women (Allen et al., 2018), and lead to disordered eating, and aspects of a negative body image such as body dissatisfaction and appearance-ideal internalization in female individuals aged 18–30 years (Wu et al., 2022). This impact might be further increased by the fact that CE-content is often also linked to body-image content (Wu et al., 2022). First studies have yielded evidence that both contents can have detrimental effects but still differ. A cross-sectional study compared the relationship between following three Instagram influencers (i.e., nutrition, fitness, and entertainment) and eating disorder (ED) symptoms and body dissatisfaction among a group of female users with a mean age of 33 years (Bocci Benucci et al., 2024). It provided evidence that while in general, increased daily use of social media, along with following nutrition and fitness accounts rather than entertainment accounts, was found to be a positive predictor of both ED symptoms and body dissatisfaction, following nutritional influencers—as opposed to fitness influencers—however, was a significant positive predictor of ED symptoms but not body dissatisfaction. In another study exposure to fitspiration content, CE content, and travel imagery on Instagram was compared by means of an online questionnaire completed by women aged between 18 and 30 years. The results revealed that engagement with fitspiration and CE material (either posted or viewed) was significantly positively associated with increased levels of compulsive exercise and athletic-ideal internalization. However, only the viewing (and not the posting) of fitspiration and CE content was significantly related to internalization of the thin ideal and symptoms of disordered eating (Wu et al., 2022). Moreover, in addition to more traditional ED symptoms, recent studies have indicated that both CE and fitspiration content (i.e., content that highlights the body ideal of a toned body) might contribute to the development of orthorexia nervosa, a disorder characterized by a compulsive fixation on healthy nutrition that is frequently associated with restrictions in other areas of life, such as loss of social contacts and health problems in individuals of both genders in adolescence and emerging adulthood (Ambwani et al., 2020; Koven and Abry, 2015). These associations were corroborated by a cross-sectional online questionnaire study that reported a higher prevalence of orthorexia nervosa symptoms in a convenience sample of social media using adult individuals who engage with CE content on Instagram (Turner and Lefevre, 2017). Furthermore, the already referenced cross-sectional study, conducted by Allen et al. (2018), found that in 17–55 year-old women who followed CE recommendations (compared to those who did not) were more likely to report higher levels of restrictive eating behavior, in the form of orthorexia nervosa. The referred findings are particularly relevant in view of the pervasiveness of these messages in the population (55% of *N* = 1,266 14-24-year-olds had heard of CE and 41% reported a willingness to try it), along with a parallel lack of knowledge about the potential harms (only 0.6% expressed skepticism, and only 18% noted both elements of healthfulness and harm; Ambwani et al., 2020).

Strongly in contrast with CE material is “foodie” content. A “foodie,” according to the Cambridge Dictionary, is a person who loves food and is very interested in different types of food—also called gourmet. Foodies show a growing presence on Instagram showcasing what they cook and what and where they eat around the world with a very hedonistic approach to food (Leer and Povlsen, 2016), often using hashtags such as #foodporn. To our knowledge, there is no study looking into the impact of consuming foodie content on mood, and body satisfaction, only preliminary evidence that the foodie content creator’s shape seems to influence the consumers’ eating intention (Jin, 2018).

In sum, there is tentative evidence from cross-sectional, observational questionnaire studies that CE content—similarly to body-image content—might be associated with negative body dissatisfaction and ED symptoms. Given that the increase in EDs and low body satisfaction is a global health problem, it is necessary to analyze factors that may contribute to this problem, such as viewing CE content. Also, given that previous research is mainly based on survey data, experimental designs with appropriate control conditions, e.g., highly hedonism-focused material such as foodie content, are needed to provide insights into causal relations (Rodgers et al., 2023; Silén and Keski-Rahkonen, 2022).

The present study aimed to explore the impact of exposure to CE vs. eating-related control content (foodie)—both with the focus on the food without depicting bodies—on Instagram with regard to affect, state body satisfaction, and food choices in a behavioral task. Additionally, we investigated whether higher-risk samples (i.e., those with high as opposed to low body dissatisfaction, ED symptoms, or orthorexic symptoms) show a stronger response when exposed to clean eating content. In a convenience sample of female university students, we therefore assessed state body satisfaction as well as positive affect (PA) and negative affect (NA) before and after exposure to content from two profiles (with a focus on clean eating (and) with foodie content; with the latter serving as the control profile) created on Instagram for the purpose of this study. After exposure to the contents, participants were offered an array of foods and told that they could choose a snack.

We hypothesized that, in a healthy female sample, viewing the CE Instagram profile would be associated with a greater increase in NA and a decrease in PA and body satisfaction, compared to viewing the foodie content Instagram profile (H1a, b and c).

Furthermore, we assumed that participants exposed to the CE profile would be more likely to choose a healthy snack than a high caloric snack compared to participants exposed to the foodie profile (H2).

And lastly, we expected that higher-risk individuals, i.e., with high scores on initial body dissatisfaction, orthorexic symptoms (with orthorexia nervosa representing a construct related to clean eating; e.g., Ambwani et al., 2020) or ED symptoms would show a greater change in PA, NA, and body satisfaction when viewing the CE profile (H3a, b and c).

## 2 Materials and methods

### 2.1 Design

This randomized experimental study used a 2 (profile: clean eating vs. foodie; between-subjects factor) × 2 (time: pre-post; within-subjects factor) design. The study was approved by the ethics committee of the University of Konstanz (reference 16/22) and was preregistered under doi: 10.17605/OSF.IO/RYUGS. The hypotheses and the analytic plan were specified prior to data collection.

### 2.2 Participants

The sample consisted of female students, mainly of psychology, recruited through SONA, the study participant platform of the University of Konstanz, and flyers distributed in the university building. Exclusion criteria included self-harm, substance dependence, other significant somatic diseases, a body mass index (BMI; kg/m^2^) lower than 17 or higher than 30, the presence of severe depressive symptoms (Patient Health Questionnaire-9, total score from items 1–8 > 14) (Gräfe et al., 2004) and acute suicidality in order to ensure that our experimental manipulation would not deteriorate any existing symptoms in highly vulnerable individuals. Further a lack of basic knowledge about using Instagram, and psychology students in their third semester or beyond were the last exclusion criteria.

Using G*Power (Faul et al., 2007), for the initially planned multivariate analysis of variance, from which we abstained while writing the preregistration, based on a medium expected effect size a power of 0.80, two groups (profiles) and who measurements (pre and post) a target sample size of *N* = 128 participants was yielded.

### 2.3 Procedure

Recruitment was conducted from April 2022 to June 2022. A total of 228 participants initially completed an online screening to check for inclusion and exclusion criteria. Eligible participants (*n* = 207) were then redirected to the landing page of the baseline survey, where they found information about the content of the study, procedure, aspects of data protection, and the voluntary nature of participation as well as the trait measures (see 2.5.1).

After completion of the survey, participants received an e-mail invitation to attend the laboratory appointment, which took place 24 h later at the earliest. From the 207 invited, 78 did not show up. In the laboratory, the remaining 129 participants signed the consent form and then completed the pre-questionnaire provided on an iPad. Subsequently, they were randomly assigned to one of the two experimental conditions (CE profile vs. foodie profile) by block randomization performed in Microsoft Excel.

In a next step, they were instructed to scroll through and inspect the respective Instagram profile on a provided smartphone (REALME C25Y, 2021) for a duration of 5 min. Two Instagram profiles were created for the study, each of which contained 16 posts and were comparable in terms of follower numbers and interactions such as likes and comments. These accounts were not accessible outside of the study. Excerpts from both profiles can be viewed in Supplementary material. No people were recognizable on the profiles; only pictures of food and its preparation were shown. These can be categorized as breakfast, preparation, drinks, main meal, to go/snack, again with an equal number per category in each profile. Participants in the CE condition viewed an Instagram profile named annalenas_cleaneatingwelt. It presented images of unprocessed food in a strategic and appealing way, based on the descriptions by Walsh and Baker (2020). Image descriptions and hashtags focused on the health aspect of certain dishes (e.g., nutrient and vitamin content, absence of industrial sugars) and were differentiated from “bad” foods (e.g., cookies, hamburgers). In addition, food preparation is increasingly addressed, which is associated with a high expenditure of time (e.g., Today I spent my morning preparing summer rolls). The storyline of the profile represents the desired path to self-improvement, which is to be achieved through clear eating rules and is conveyed as desirable. The second profile, annalenas_foodiewelt, served as the control foodie content profile and depicted images of different foods, recipes, and restaurants, sharing beautiful moments of culinary life, without information about restrictions or ingredients. Instead, the focus was placed on the taste and enjoyment of the dishes (e.g., cake simply makes you happy), as well as the sociability aspect (e.g., cooking evening with friends, restaurant visits). The pictures were snapshots of the food, the dishes were not specially prepared for the photos. The storyline presents a person who wants to share the beautiful moments of culinary life with the help of her account.

During the condition, their self-determined on-screen activities (scrolling through the posts at their own pace) were recorded on the smartphone via a screen capture program (these data are beyond the scope of the present manuscript and will be published elsewhere; see exploratory analyses in preregistration). After the profile viewing, participants were asked to complete the post-questionnaire and told that as a thank you for their participation, they could choose a snack from an array of food consisting of healthy snacks (apples, pears, bananas) and high caloric foods (Ferrero hanuta, wafer filled with hazelnut cream, vegan hanuta, or SNICKERS).

### 2.4 Measures

#### 2.4.1 Trait measures

In the following, all measures used in the present study are presented in alphabetical order. Other measures employed in the project are outlined in the preregistration. Additionally, we queried hunger ratings (0–10) as well as time since last meal in hours at the beginning of the survey.

Düsseldorf Orthorexia Scale (DOS) (Barthels et al., 2015). The DOS is a self-report measure of orthorexic behavior comprising 10 items rated on a four-point Likert scale ranging from “4” (this applies to me) to “1” (this does not apply to me). Higher scores indicate higher levels of orthorexic behavior. The scale showed acceptable internal consistency in the present sample (Cronbach’s *α* = 0.77).

Eating Disorder Examination Questionnaire (EDE-Q) (Fairburn and Beglin, 1994; Hilbert et al., 2016). The EDE-Q measures the range and severity of ED symptoms. It consists of 28 items that can be summarized into a global scale and four subscales (Restraint, Eating Concern, Shape Concern, and Weight Concern). In the present sample, we combined the subscales Weight and Shape Concern as a proxy for body dissatisfaction, as well as the subscales Restraint and Eating Concerns as a proxy for ED symptoms (beyond body dissatisfaction). Internal consistency in the present study was excellent for the total score (Cronbach’s *α* = 0.95) and high for the two combined subscale scores (Cronbach’s α = 0.93).

#### 2.4.2 State measures

Body Image States Scale (BISS) (Cash et al., 2002; German-language version Quittkat et al., personal communication). The BISS measures individuals’ self-evaluation of and affect regarding their physical appearance, with six statements rated on a 10-point Likert scale from “1” (extremely dissatisfied) to “9” (extremely satisfied). In the present study, internal consistency was good to very good (pre-measurement: Cronbach’s α = 0.87; post-measurement: α = 0.91).

Positive and Negative Affect Schedule (PANAS) (Watson et al., 1988; German-language version: Breyer and Bluemke, 2016). The PANAS measures positive and negative affect with 10 items each, rated on five-point scale from “1” not at all to “5” very much. The internal consistency of the PANAS in the present sample was acceptable to good (pre-measurement: α = 0.78; post-measurement α = 0.81).

### 2.5 Data analyses

Data analysis was performed using SPSS Statistics (version 28; IBM Corp, 2021). Descriptive analyses (frequencies, means, and standard deviations) are provided for the demographic description of the sample. To test for significant differences between participants in the two conditions (those viewing CE vs. foodie content) regarding age, BMI, presence of ED symptoms (EDE-Q) and mental health (PHQ-9), we conducted t-tests for independent samples. To analyze the impact of the different profiles on affect (PA and NA) and body satisfaction (H1a-c), we conducted three 2 (time) × 2 (profile) mixed analyses of variance (ANOVAs). A Chi-square test was conducted to test for significant differences between the randomized groups and the food choice (healthy vs. high caloric caloric) (H2). Subsequently, the impacts of baseline body satisfaction (combined EDE-Q Shape and Weight Concern scales), ED symptoms (combined EDE-Q Restraint and Eating Concern scales), and orthorexic symptoms (DOS) on change in PA, NA, and state body satisfaction (pre-post) in the group exposed to CE content were examined making use of three multiple linear regression analyses (H3a-c). The latter choice of data analysis method is in contrast to the one proposed in the preregistration but is more suitable.

## 3 Results

The final sample comprised 129 women (CE profile: *n* = 64; “foodie” profile: *n* = 65). The two groups did not differ regarding age, BMI, ED symptoms (EDE-Q total, combined restraint and eating concern and combined shape and weight concern scales, respectively), orthorexic symptoms, or general psychopathology (PHQ-9) (Table 1). Furthermore, the two groups did not differ in hunger ratings as well as the time since their last meal (both *p* > 0.10). A post-hoc power analysis revealed that in the conducted ANOVAs with this sample size, a power of 0.80 and an error probability of 0.05, we were able to detect small to medium effect sizes of *f* = 0.12 in the main analyses.

**Table 1:** Group differences in demographic and clinical characteristics.

Variables	Clean eating			Foodie			Test	
	*M*	*SD*	Range	*M*	*SD*	Range	*t*	*p*
Age	21.28	2.09	10.00	21.34	1.98	9.00	−0.16	0.87
BMI	21.43	2.23	10.45	21.90	2.84	12.18	−1.06	0.29
EDE-Q total	2.50	1.20	5.14	2.22	0.91	4.32	1.63	0.11
EDE-Q WC and SC	2.86	1.47	5.64	2.60	1.17	5.06	1.11	0.27
EDE-Q RS and EC	1.88	0.91	4.40	1.64	0.63	3.30	1.77	0.08
PHQ-9	1.55	0.38	1.33	1.45	0.26	1.00	1.49	0.14
DOS	1.77	0.40	1.60	1.74	0.42	1.80	0.42	0.68

CE, Clean Eating; BMI, body mass index; PHQ-9, mean Patient Health Questionnaire-9; EDE-Q total, mean Eating Disorder Examination Questionnaire total score; EDE-Q WC and SC, mean EDE-Q combined weight and shape concern scale; EDE-Q RS and EC, mean EDE-Q combined restraint and eating concern scale; DOS, Düsseldorf Orthorexia Scale.

### 3.1 Impact of profile exposure on positive and negative affect and body satisfaction

**Figure 1:**
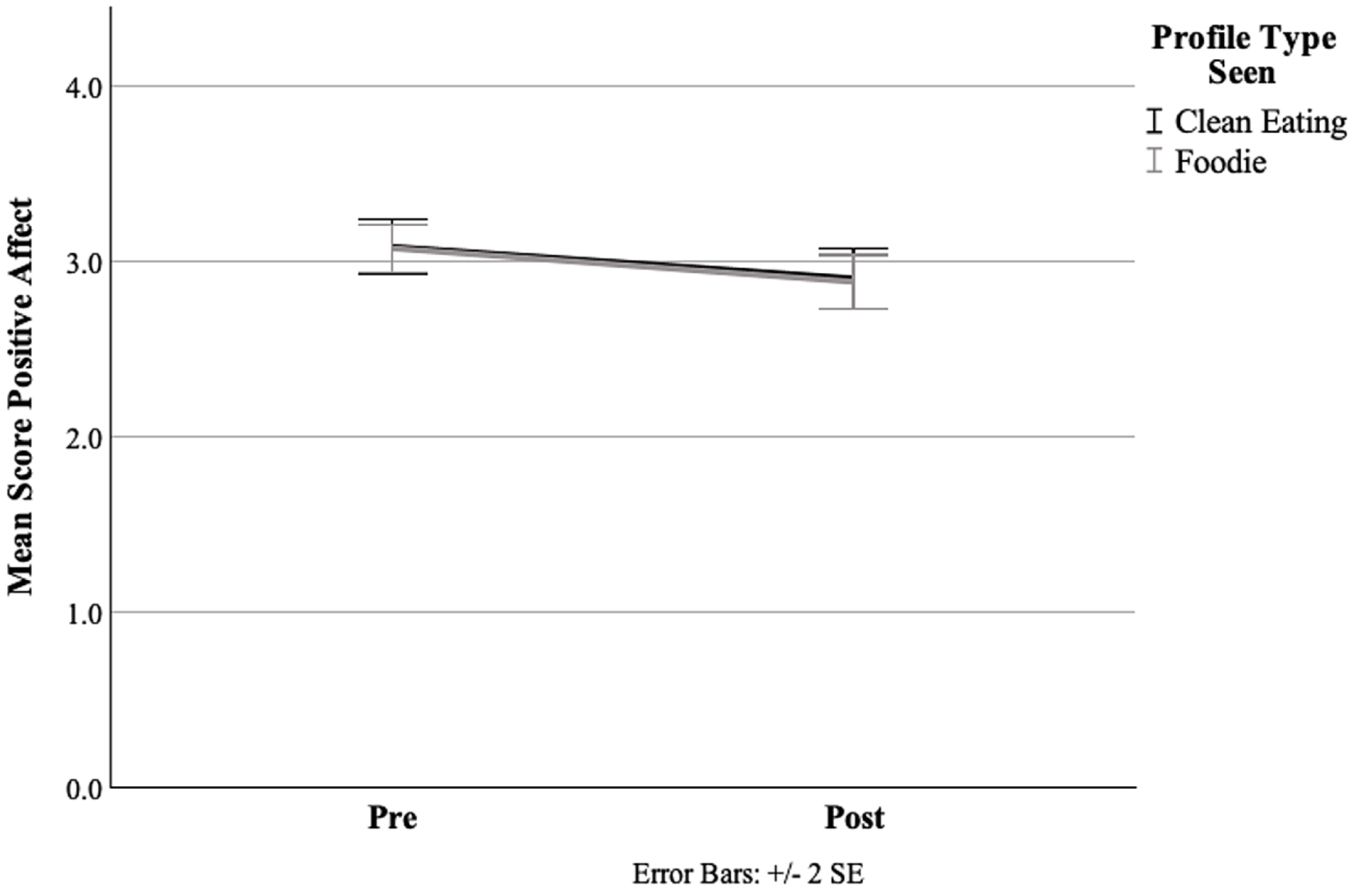
Means of positive affect (PA) for both profile conditions from pre to post, measured using the PANAS.

Regarding PA, in the repeated measures ANOVA, there was no significant time × profile interaction [*F*(1, 127) = 0.03, *p* = 0.86, _p_𝜂^2^ < 0.00] and no significant main effect of profile [*F*(1, 127) = 0.02, *p* = 0.90, _p_𝜂^2^ < 0.01]. Thus, the hypothesis 1a concerning PA was not confirmed. However, there was a significant main effect of time [*F*(1, 127) = 33.99, *p* < 0.01, _p_𝜂^2^ = 0.21], with a reduction in PA from before to after exposure across groups (Figure 1). Table 2 shows the pre-test and post-test means and standard deviations of the dependent variables for the CE group and the foodie group.

**Table 2:** Means and standard deviations for dependent variables per profile before and after profile viewing.

Variables	Timepoints	Clean eating		Foodie	
		*M*	*SD*	*M*	*SD*
Positive affect	Pre	3.08	0.63	3.08	0.54
	Post	2.90	0.68	3.08	0.68
Negative affect	Pre	1.44	0.51	1.33	0.46
	Post	1.43	0.48	1.23	0.36
Body satisfaction	Pre	3.86	1.39	3.68	0.97
	Post	4.10	1.55	3.65	1.01

**Figure 2:**
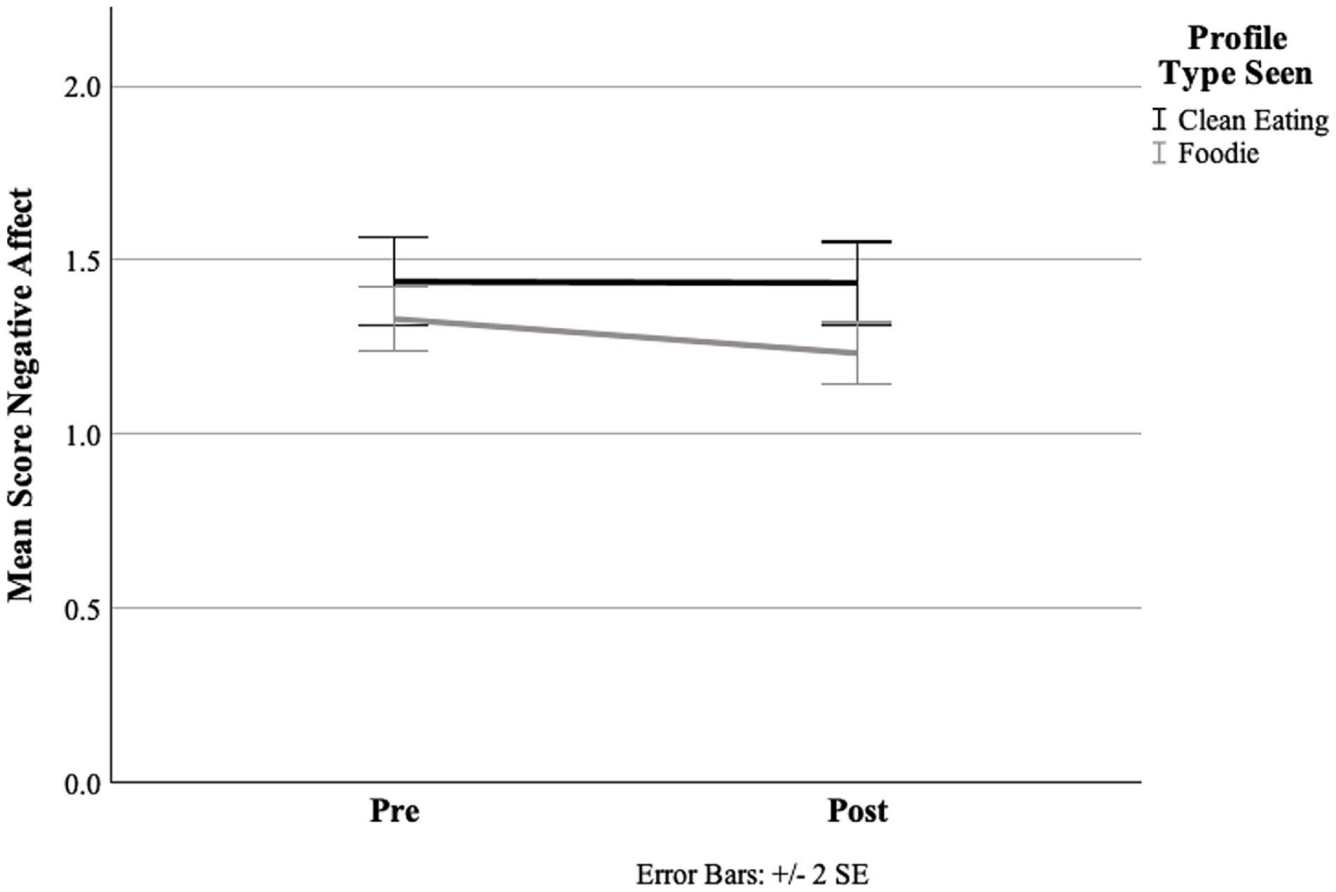
Means of negative affect (NA) for both profile conditions from pre to post, measured using the PANAS.

With regard to NA, in the repeated measures ANOVA, we found a significant interaction of time × profile [*F*(1, 127) = 4.32, *p* = 0.04, _p_𝜂^2^ = 0.03]; considering the simple main effects, this resulted in a significant reduction in NA from pre- to post-measurement for participants viewing the “foodie” profile [*F*(1, 65) = 0.02, *p* < 0.01, _p_𝜂^2^ = 0.21]. As illustrated in Figure 2, no significant changes emerged for the CE profile [*F*(1, 64) = 0.01, *p* = 0.94, _p_𝜂^2^ = < 0.01]. Thus, hypothesis 1b regarding NA was partially confirmed. Both main effects were also significant [time: *F*(1, 127) = 4.91, *p* = 0.03, _p_𝜂^2^ = 0.04; profile: *F*(1, 127) = 4.56, *p* = 0.04, _p_𝜂^2^ = 0.04].

**Figure 3:**
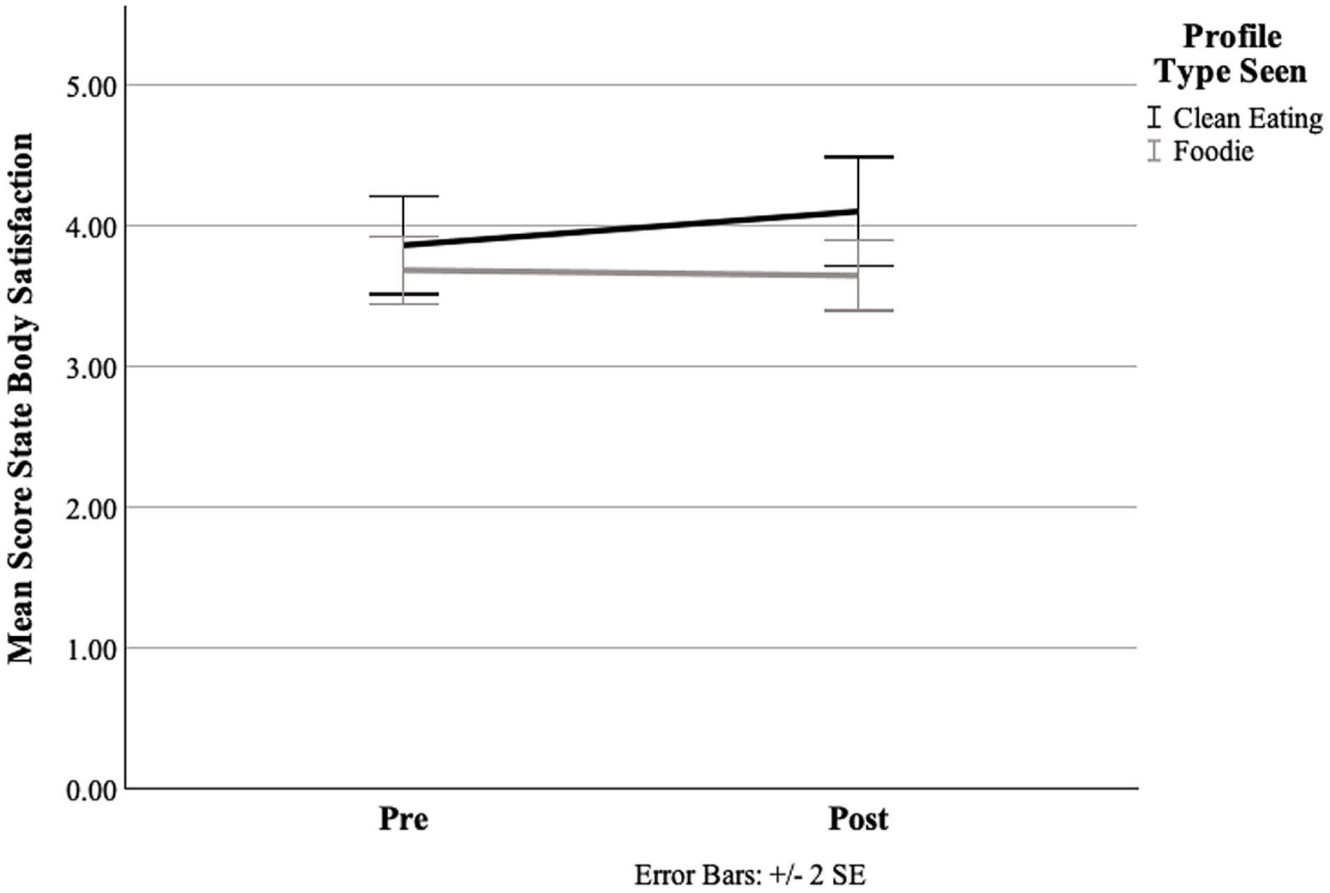
Means of body satisfaction accordingly: for both profile conditions from pre to post, measured using the BISS. Note: State body satisfaction assessed using the BISS (Body Image States Scale), with higher scores indicating lower body satisfaction.

Regarding body satisfaction, it should be noted that higher scores on the BISS indicate lower body satisfaction (Figure 3). The repeated measures ANOVA revealed a significant interaction effect of time × group [*F*(1, 127) = 9.02, *p* < 0.01, _p_𝜂^2^ = 0.07]. Regarding simple main effects, in neither profile was there a significant change in body satisfaction over time [CE: *F*(1, 65) = 0.45, *p* = 0.50, _p_𝜂^2^ < 0.01; “foodie”: *F*(1, 65) = 0.027, *p* = 0.61, _p_𝜂^2^ < 0.01] that substantiated this interaction effect. Thus, an initial confirmation of hypothesis 1c regarding body satisfaction needs to be discarded. While there was a significant effect of time across profiles [*F*(1, 127) = 4.72, *p* = 0.03, _p_𝜂^2^ = 0.04], no significant main effect emerged with respect to the CE profile [*F*(1, 127) = 2.14 *p* = 0.15, _p_𝜂^2^ = 0.02].

### 3.2 Group differences regarding food choice (healthy vs. high caloric)

In the CE group 13 participants (20.31%) chose a healthy food, in contrast to 49 participants (76.56%) who chose a high caloric snack, while two participants did not accept either food offered. In the foodie group, 16 participants (25%) chose a healthy food while the remaining 48 individuals (75%) chose high caloric snacks. A chi square test revealed that the groups did not differ with respect to their choice of snack, χ^2^(2) = 0.25, *p* = 0.99, thus hypothesis 2 is not confirmed.

### 3.3 Impact of viewing the CE profile depending on pre-existing body dissatisfaction, eating disorder, and orthorexic symptoms

Regression analyses showed, that none of the three pre-existing symptom groups significantly predicted the change in PA (all standardized *β* < |0.42|, all *p* > 0.11), NA (all standardized β < |0.29|, all *p* > 0.29), or body satisfaction (all standardized β < |0.44|, all *p* > 0.09) over the course of CE profile viewing. Thus, hypotheses 3a–c were not confirmed.

## 4 Discussion

### 4.1 Impact of the two profiles clean eating and “foodie” on affect and body satisfaction

One aim of the present study was to determine the impact of CE Instagram content on PA and NA as well as body satisfaction in a sample of young women. Specifically, we hypothesized that viewing a CE profile would negatively influence affect and body satisfaction as compared to viewing a foodie profile. With regard to PA, we did not find any difference between the two conditions CE and foodie. It is important to notice that a significant decrease in PA emerged for both profiles, as a result of using Instagram, which corresponds to previous research on Instagram and its potential detrimental effects in regarding mental health (for a systematic overview: Faelens et al., 2021). Regarding NA, an interaction effect was observed insofar as NA decreased in the foodie condition but remained unchanged in the CE condition. One reason for this finding might lie in the focus of the respective Instagram captions, with the focus on self-compassion and pleasure in the foodie condition potentially leading to a reduction in NA, as reported in an experimental study by Slater et al. (2017). This study found that female students who were subjected to viewing fitspiration images but with self-compassion captions reported a higher level of body appreciation and a reduction in negative mood than those who viewed fitspiration images alone. This result suggests that self-compassion might offer a new avenue to mitigate the negative impact of social media on women’s body satisfaction. The aspect of captions related to self-compassion should also be investigated further with regard to CE content. Therefore, in our study, the significant reduction in NA in the foodie condition could result from the pleasure and self-compassion effect triggered by the images of foods considered to be appetizing. However, contrary to our expectation, NA did not increase in response to the CE profile. This might be explained by the fact that our hypothesis derives from the literature on fitspiration. However, while fitspiration is often associated with CE (Tiggemann and Zaccardo, 2018), the content is not the same as the study by Bocci Benucci and colleagues, referred to above, has shown. Moreover, these observed effects were evident despite the short period of use of only five minutes, even though users usually spend much longer on Instagram and with greater frequency (Auxier and Anderson, 2021). According to the results of our study, it can be concluded that CE profiles maintain NA whereas foodie profiles can lower NA and might have a certain protective effect.

In terms of body satisfaction, our findings indicated a possible negative association between CE exposure and body satisfaction, however, the simple main effects did not substantiate the interaction. The initial finding of an interaction effect, however, supports previous cross-sectional questionnaire studies that reported an association between CE and restrictive diets, which in turn have been associated with lower body satisfaction (Allen et al., 2018; Wu et al., 2022). Moreover, our study is the first to report very tentative evidence of a causal relationship between viewing CE content and a reduction in body satisfaction. Thus, if this result is further confirmed, it shows that merely depicting a presumably healthy lifestyle, without even showing actual bodies, is sufficient to affect body satisfaction. This finding might be explained by the apparent - potentially implicit - association between CE and thin and toned bodies as seen in fitspiration content.

### 4.2 Influence of clean eating content exposure on food choices

Our hypothesis that displaying the CE profile would lead to healthier food choices than displaying the foodie profile was not supported by the present results. In fact, participants in the CE group did not tend to choose healthy foods. The analysis showed that in the overall sample, most participants chose high caloric foods. This might be due to the fact that the high caloric snacks are easier to share with others during meals and are more hygienic as they are packaged. It would be worth investigating whether food choices depend on the situation and are moderated by more practical and social aspects. Another explanation could also depend on the short exposure time to the CE profiles that the study participants were exposed to. Considering Sobal and Bisogni (2009) hypothesis that food choices result from three different factors: life events and experiences, exposure to cultural and social factors, and personal value system, it would be plausible to analyze these factors using longitudinal studies to understand whether, and if so, when and under which conditions the content shown on Instagram is internalized and translated into behavioral choices.

### 4.3 The prediction of change in negative affect and state body satisfaction when consuming clean eating content by initial body satisfaction, eating disorder symptoms, and orthorexic symptoms

We further hypothesized that ED symptoms and initial body satisfaction might be predictive for the changes in NA, PA, and state body satisfaction in participants viewing the CE profile. However, the results did not support this hypothesis. Several questionnaire-based studies have demonstrated an association of the exposure to fitspiration content with ED symptoms and affect (Griffiths et al., 2018; Hung, 2022). Only one previous study, conducted by Krug et al. (2020), has examined these associations in a more situational context using an experimental ecological momentary assessment design examining the effect of fitspiration images on body image, affect, and eating behavior in a sample of women. The results revealed that while the perceived pressure to achieve an ideal body was significantly higher for participants who were exposed to fitspiration as compared to neutral images, there were no differential impacts on body image, affect, and eating behavior. These findings are in line with the results of the present study. However, as there is no other research on the exposure to CE profiles, it is not possible to draw any definitive conclusions.

Lastly, our study did not reveal a prediction of change in NA, PA, and body satisfaction from pre- to post-CE content exposure by initial orthorexic symptoms. Regarding the lack of change in affect, it might be hypothesized that in individuals with orthorexic symptoms, the sight of clean food and CE contents might represent a source of reassurance and may be associated with the idea of a healthy body and a lower risk of health problems. In terms of body satisfaction, the lack of significant changes in state body satisfaction might be based on the fact that individuals with high levels of orthorexic symptoms show an obsession with the nutritional quality of food and restrictive control over food intake, but often in the absence of a desire for a thin and toned body (Koven and Abry, 2015), comparable to those with low levels of orthorexic symptoms. Therefore, viewing these foods does not trigger a comparison regarding the body and consequently does not lead to negative comparisons, as proposed in the tripartite influence model (Thompson et al., 1999).

### 4.4 Limitations

Several limitations of the present study should be mentioned. First, the student sample consisted exclusively of women, which may limit the generalizability of the findings. However, we chose to focus on women as evidence shows that EDs are more prevalent among women, they are more influenced by body-related content compared to men (Andersen and Ryan, 2009), and most previous studies examined female samples (for an overview: Roorda and Cassin, 2025; Wu et al., 2024) which allowed for a better comparison. Second, participants were only exposed to the CE contents for a short period of time, and it is possible that potential effects only become apparent after a longer period. Third, it was not possible to verify the effects over time since participants only viewed the contents once. Lastly, despite a considerable sample size at the outset, the subgroup analysis in the CE group, showed a significantly reduced power.

## 5 Conclusion and implications

This randomized experimental study examined the short-term effects of exposure to *clean eating* (CE) versus foodie Instagram content on affective states, body satisfaction, and food choice behavior in a sample of young adult women. It further explored whether individual differences in body dissatisfaction, ED symptoms, or orthorexic tendencies moderated participants’ psychological and behavioral responses to CE content. Findings indicated a significant overall reduction in PA across both conditions, suggesting that Instagram use per se is associated with declines in mood and emotional wellbeing. However, negative NA significantly decreased following exposure to foodie content but not CE content, suggesting that content emphasizing enjoyment and social connection may have a more favorable impact on mood than health-restrictive narratives. Although no statistically significant change in body satisfaction emerged as a simple main effect, the observed time-by-condition interaction suggests a potential adverse influence of CE exposure on body image. Notably, food choice behavior did not differ between groups, and no moderation effects were observed based on baseline vulnerabilities. These null effects may be attributable to the brief exposure duration or to the subtler nature of CE messaging compared to overtly appearance-focused content such as fitspiration. From a theoretical implication standpoint, these findings underscore the psychological relevance of CE content, which, despite its health-promoting appearance, may contribute to the internalization of rigid dietary ideals and diminished body satisfaction.

From a practical standpoint, these findings highlight the importance of integrating CE-related content into psychoeducational and media literacy initiatives designed to prevent body dissatisfaction and EDs. Interventions should encourage critical reflection on food-related content that implicitly promotes moral hierarchies of eating and self-worth through dietary discipline. Clinicians may consider including questions about CE content exposure in intake assessments and treatment planning for clients with body image concerns or restrictive eating behaviors. Furthermore, public health campaigns, educators, and content creators should be encouraged to disseminate balanced, inclusive, and emotionally supportive representations of food that prioritize psychological wellbeing.

Future directions for research include a replication of the present study using a larger and more diverse sample and to evaluate the effects of prolonged CE content over time. Future studies might also employ longitudinal and ecologically valid designs to assess the cumulative impact of CE exposure, include direct comparisons with other social media trends (e.g., fitspiration), and examine diverse populations, potentially also clinical, to improve generalizability. Additionally, it would be worthwhile to look into the association of affect and food preference, or control for this aspect in future studies.

In sum, this experimental study provided first insights into the short effects of one-time consumption of CE content on social media on affect but not body satisfaction and food choice. It did no yield evidence regarding a higher susceptibility of at-risk individuals high in ED or orthorexic symptoms, respectively, or body dissatisfaction. The findings suggest that both the dissemination and influence of CE content on social media should be subject to more systematic observation in the future.

## Data Availability

The raw data supporting the conclusions of this article will be made available by the authors, without undue reservation.
