# Supplementary material for: Food and mood: how clean eating content on social media influences affect and body satisfaction in women
Source: Front Psychol. 2025 Sep 22;16:1531142. doi: 10.3389/fpsyg.2025.1531142 (PMC12497578; doi:10.3389/fpsyg.2025.1531142)

**Instagram profiles**

The screenshots below show excerpts from the Instagram profiles at the end of the data collection (03.06.2022). Both profiles can be accessed directly via Instagram at the following URLs: https://www.instagram.com/annalenas_cleaneatingwelt/; https://www.instagram.com/annalenas_foodiewelt/. The complete photo sets can be requested from the authors. License-free images from the Internet were included in the profile design. The privately produced photographs are subject to the copyright of the authors.

**A1. Clean eating profile**


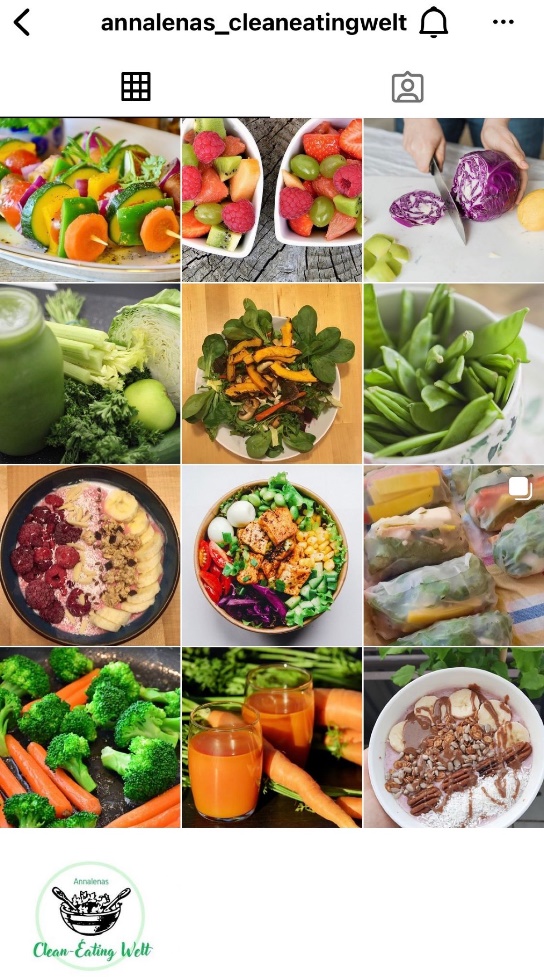

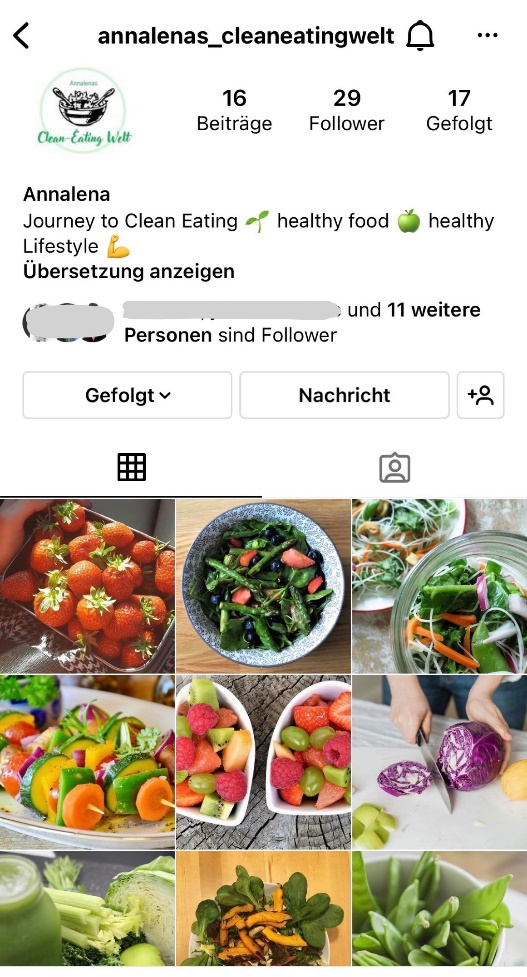


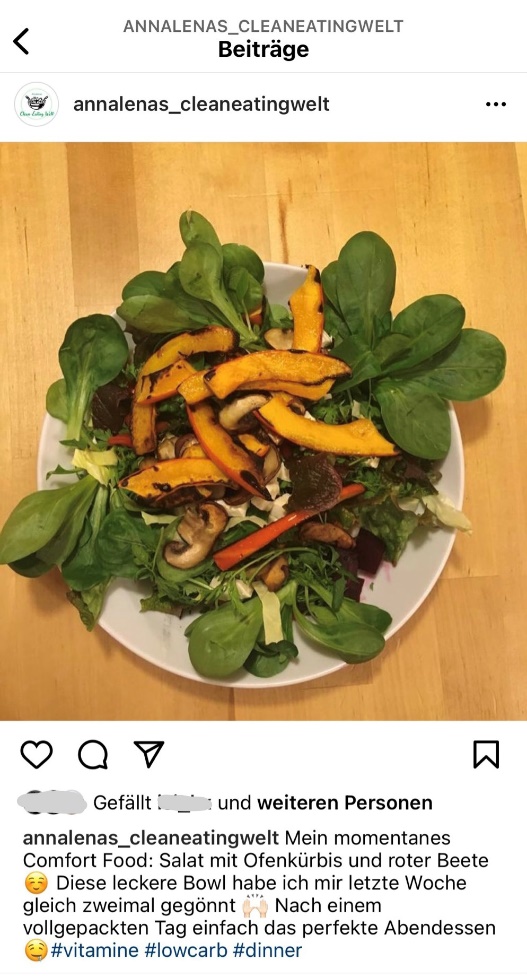
**
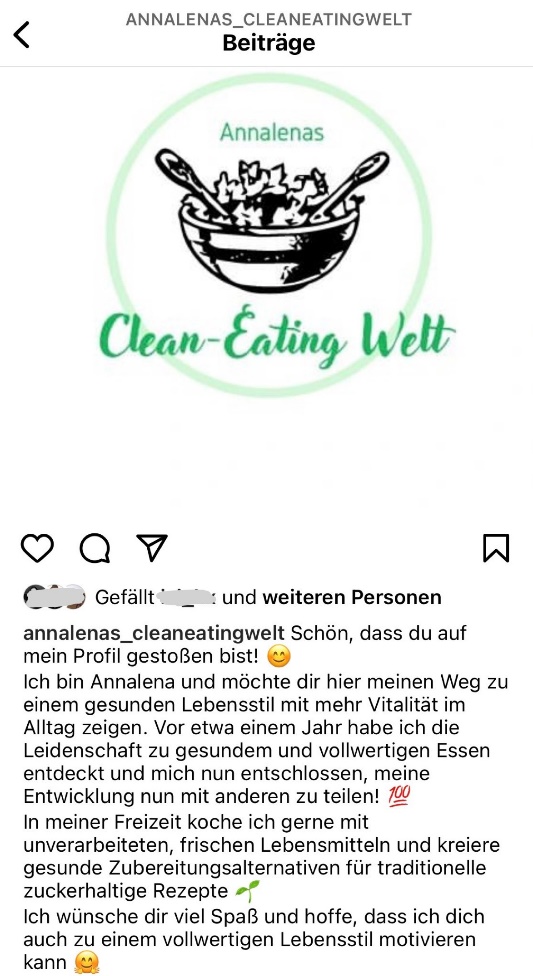
**
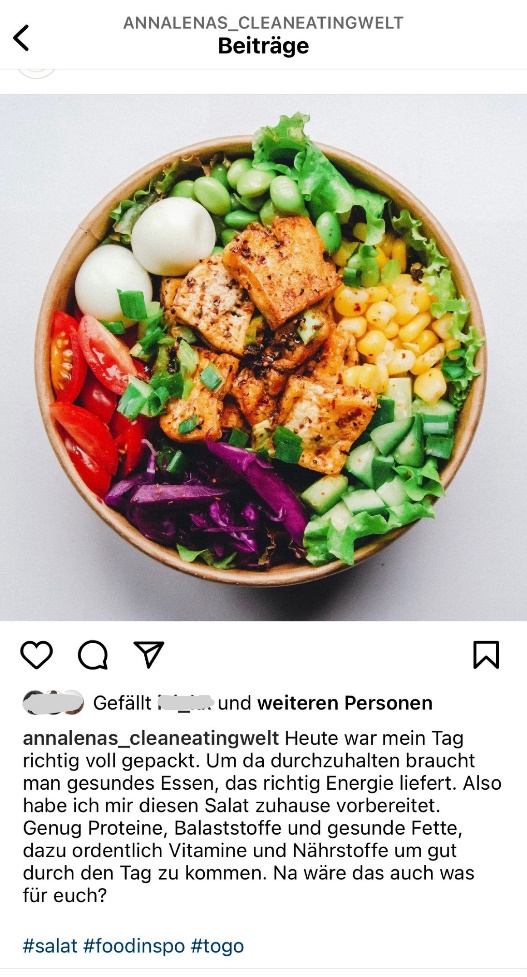

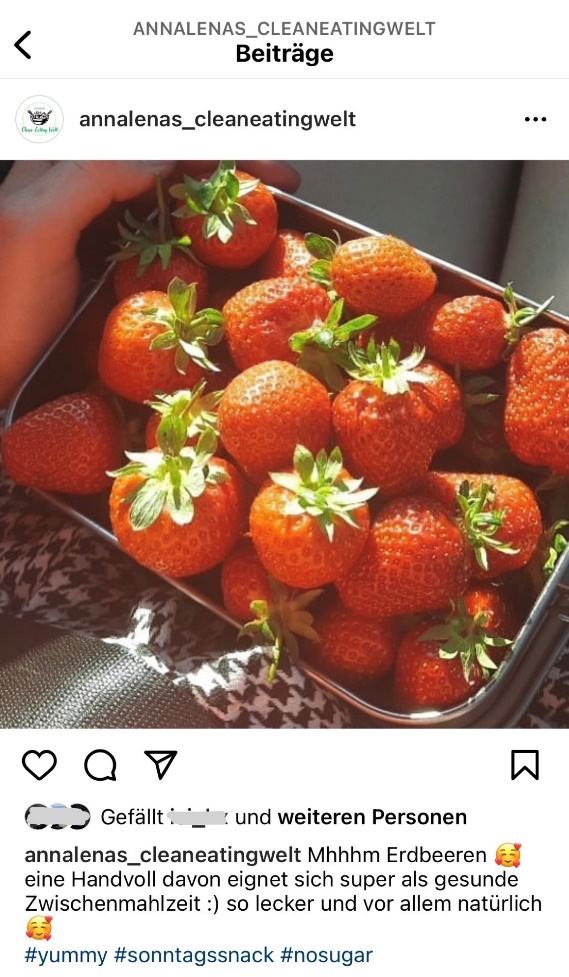


**A2. Foodie profile**


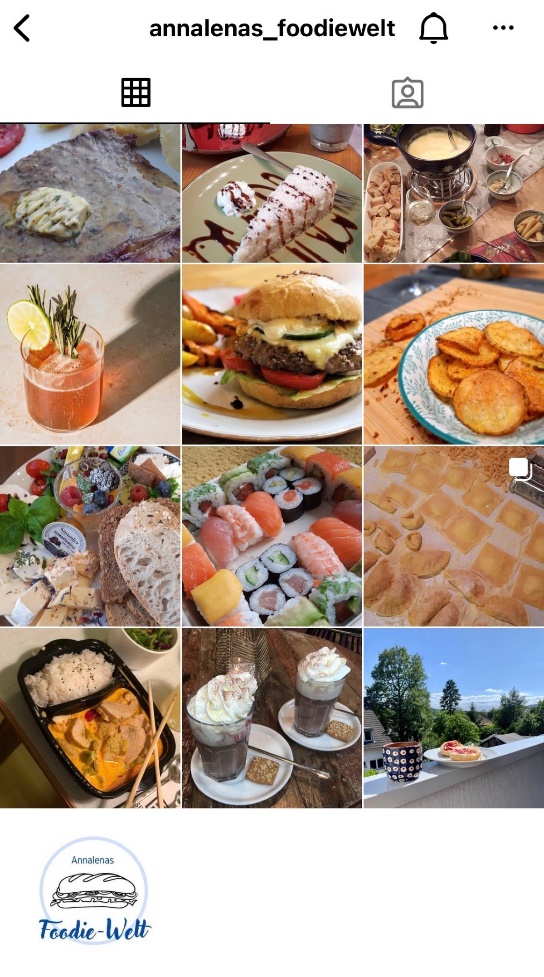

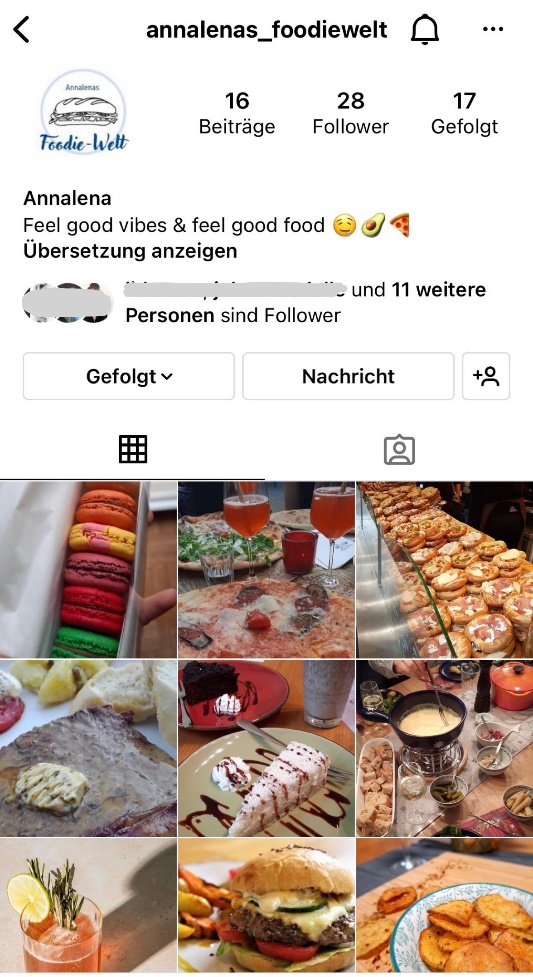

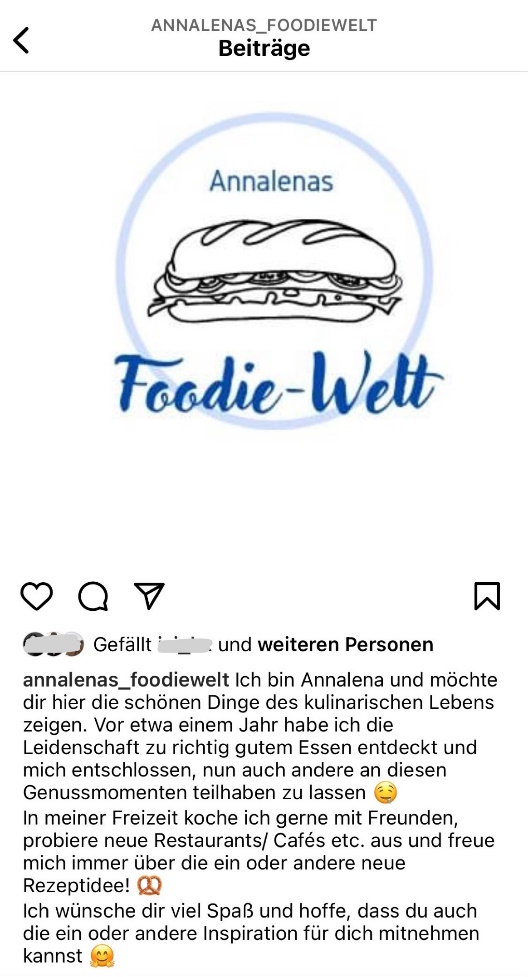

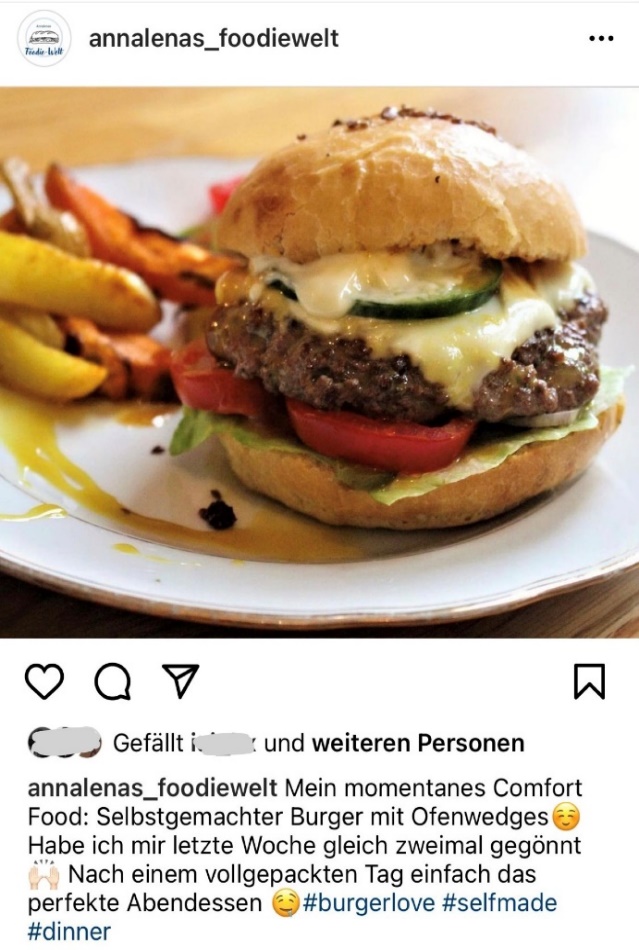


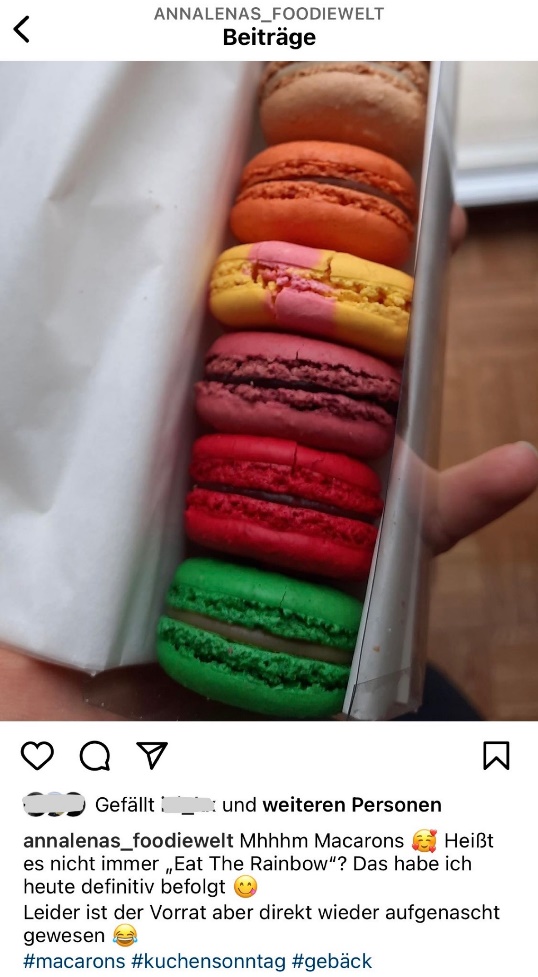

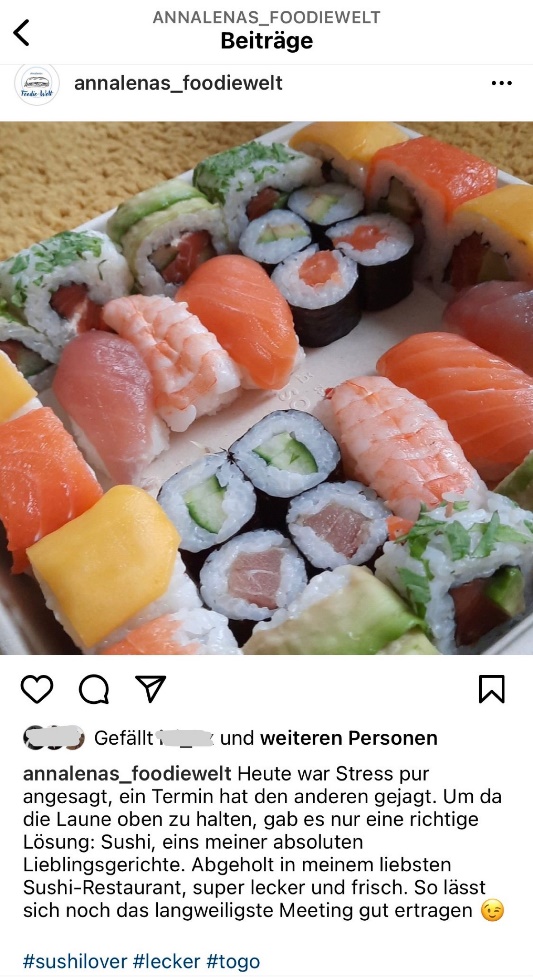

Supplement: Supplementary file 2 [file Table_1.DOCX]
